# Case report: Withdrawal of angiotensin-converting enzyme inhibitors in children with advanced chronic kidney disease and rapidly declining kidney function

**DOI:** 10.3389/fped.2023.1172567

**Published:** 2023-05-04

**Authors:** Tomáš Seeman, Jiří Dušek

**Affiliations:** ^1^ Department of Pediatrics, University Hospital Ostrava, Ostrava, Czechia; ^2^ Department of Pediatrics, 2nd Medical Faculty, Charles University Prague, Prague, Czechia; ^3^ Faculty of Mediciny, University of Ostrava. Ostrava, Czechia

**Keywords:** estimated glomerular filtration rate (eGFR), blood pressure (BP), proteinuria, case report, chronic kidney disease

## Abstract

**Background:**

It is not known whether withdrawal of angiotensin-converting enzyme inhibitors (ACEIs) in children with advanced chronic kidney disease (CKD) is beneficial similar to adults. We report a case series of children with advanced CKD whose ACEIs were stopped.

**Methods:**

In the last 5 years, we stopped ACEIs in seven consecutive children on ACEI therapy with rapidly declining CKD stage 4–5. The median age was 12.5 years (range 6.8–17.6); the median estimated glomerular filtration rate (eGFR) at stopping ACEIs was 12.5 ml/min/1.73 m^2^ (range 8.8–19.9).

**Results:**

Six to twelve months after stopping ACEIs, the eGFR increased in five children (71%). The median absolute increase of eGFR was 5.0 ml/min/1.73 m^2^ (range −2.3 to +20.0) and relative increase of eGFR was 30% (range −34 to +99). The median follow-up after stopping ACEIs was 2.7 (range 0.5–5.0) years, either until the start of dialysis (*n* = 5) or until the last follow-up without dialysis (*n* = 2).

**Conclusions:**

This case series showed that withdrawal of ACEIs in children with CKD stage 4–5 and rapidly declining kidney function may lead to an increase in eGFR.

## Introduction

Treatment of children with chronic kidney disease (CKD) with renin–angiotensin–aldosterone system inhibitors (RAASi) is the cornerstone of conservative therapy due to their antihypertensive, antiproteinuric, and renoprotective effects. Recently, van den Belt et al. (1) published a retrospective study on the effects of discontinuation of RAASi in children with advanced CKD. Withdrawal of RAASi led to a more rapid estimated glomerular filtration rate (eGFR) decline than before discontinuation. This finding was in sharp contrast to several previous similar trials in adults. These studies showed that stopping of RAASi leads to improvement of eGFR in the majority of patients (2–4). In the largest adult study, Ahmed et al. demonstrated increased eGFR in 60% of adults with advanced CKD and undoubtedly delayed onset of kidney replacement therapy (KRT) in the majority of patients after stopping inhibitors of the RAASi (2). The reasons for the discrepancy between the result of pediatric and adult studies are not fully clear and need further analyses.

We aimed to present a case series of seven children from our tertiary pediatric nephrology center with CKD stage 4–5 (CKD5) and rapidly declining GFR with stopping angiotensin-converting enzyme inhibitors (ACEIs). Our case series is unique as no pediatric study has yet shown that, in contrary to adults, withdrawal of ACEIs in children with CKD5 can be beneficial in terms of kidney function.

## Case description

In the last 5 years, we stopped ACEIs in all seven consecutive children on therapy with RAASi who had rapidly declining GFR toward CKD stage 5 and have been not yet indicated for KRT with the aim to win time for preparation of KRT. We did it after reading the positive results of stopping ACEI in adults with advanced CKD (2–4) in hope to delay onset of KRT.

The median age of the patients was 12.5 years (range 6.8–17.6); the primary kidney diseases were mainly congenital anomalies of kidneys and urinary tract. Five children received ramipril (median dose 2.3 mg/m^2^/day) and two received enalapril (0.1 and 0.2 mg/kg/day). The indication for ACEI was hypertension (*n* = 5) and hypertension with proteinuria (*n* = 2). The median eGFR [Schwartz formula (5)] at stopping ACEI was 12.5 ml/min/1.73 m^2^ (range 8.8–19.9), and six children were already in CKD stage 5. The median eGFR slope 6–12 months before stopping ACEI was −9.8 ml/min/1.73 m^2^/year (range from −4.0 to −19.5). All children had declining eGFR at least in the last two assessments before stopping ACEI and had no acute infection or dehydration. The blood pressure index was calculated as patients’ blood pressure (BP) divided by the 95th percentile. Formal statistical analysis was not performed due to the low number of cases.

## Diagnostic assessment and therapeutic intervention

### Kidney function

**Figure 1:**
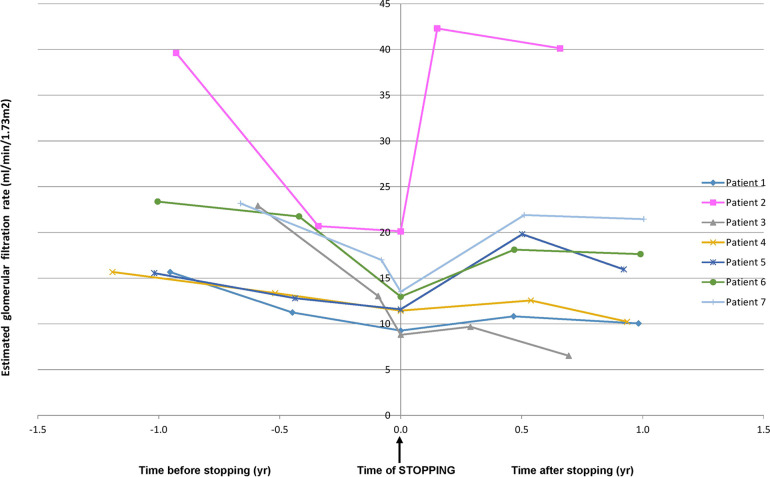
Glomerular filtration rate slope in all individual patients before and after stopping ACEIs. ACEIs, angiotensin-converting enzyme inhibitors.

Six to twelve months after stopping ACEIs, the eGFR increased in five of seven children (71%). The median absolute increase of eGFR was 5.0 ml/min/1.73 m^2^ (range −2.3 to +20.0) and relative increase of eGFR was 30% (range from −34 to +99). Improvement of eGFR >25% from baseline value [a parameter used by Ahmed et al. in their study (2)] occurred in 57% of children. The eGFR slope in all individual patients is given in Figure 1. The median follow-up after stopping ACEI was 2.7 (range 0.5–5.0) years, until the start of KRT (*n* = 5) or until the last follow-up without KRT (*n* = 2). The prognosis of these children improved as the time without the need for KRT (dialysis/transplantation) was prolonged.

### Blood pressure, proteinuria, and serum potassium

The median BP and proteinuria at the time of stopping ACEI were 107/68 mmHg (BP index 0.93) and 28 mg/mmol creatinine (range 13–41), respectively, and they increased to 121/80 mmHg (BP index 0.97) and 138 mg/mmol creatinine (range 43–209) after stopping, respectively. The median serum potassium was 5.3 mmol/L (range 4.9–6.7) at the time of stopping ACEI and 4.8 mmol/L (range 4.4–5.2) after stopping. Patient and laboratory characteristic in all individual patients is given in Table 1.

**Table 1:** Patient and laboratory characteristics in all individual patients before and after stopping ACEIs.

	All patients (*n* = 7)	Responders (any increase of eGFR 6–12 months after ACEI cessation) (*n* = 5)	Non-responders (no increase of eGFR 6–12 months after ACEI stopping) (*n* = 2)
Age (years)	12.5 (6.8–17.6)	13.5 (10.3–17.6)	10.1 (6.8–13.4)
Underlying kidney disease	ARPKD (*n* = 3), NPHP (*n* = 2), PUV, RN	ARPKD (*n* = 2), NPHP, PUV, RN	ARPKD, NPHP
Observation period after ACEI cessation (months)	Pt 1: 12	Pt 1: 12	
	Pt 2: 7	Pt 2: 7	
	Pt 3: 8, Pt 4: 10	Pt 5: 10	
	Pt 5: 10	Pt 6: 12	Pt 3: 8
	Pt 6: 12, Pt 7: 6	Pt 7: 6	Pt 4: 10
	**Median: 9 (6–12)**	**Median: 9**	**Median: 9**
eGFR at ACEI cessation (ml/min/1.73 m^2^)	Pt 1: 9	Pt 1: 9	
	Pt 2: 20	Pt 2: 20	
	Pt 3: 9, Pt 4: 11	Pt 5:12	
	Pt 5:12	Pt 6: 13	Pt 3: 9
	Pt 6: 13, Pt 7: 14	Pt 7: 14	Pt 4: 11
	**Median: 12 (9–20)**	**Median: 14**	**Median: 10**
Pre-ACEI cessation eGFR slope (ml/min/1.73 m^2^/12 months)	Pt 1: −6	Pt 1: −6	
	Pt 2: −20	Pt 2: −20	
	Pt 3: −14, Pt 4: −4	Pt 5: −4	
	Pt 5: −4	Pt 6: −10	Pt 3: −14
	Pt 6: −10, Pt 7: −10	Pt 7: −10	Pt 4: −4
	**Median: 10 (−4 to −20)**	**Median: −10**	**Median: −9**
Post-ACEI cessation eGFR slope (ml/min/1.73 m^2^/12 months)	Pt 1: 1, Pt 2: 20	Pt 1: 1	
	Pt 3: −2, Pt 4: −1	Pt 2: 20	
	Pt 5: 4	Pt 5: 4	Pt 3: −2
	Pt 6: 5, Pt 7: 8	Pt 6: 5, Pt 7: 8	Pt 4: −1
	**Median: 5 (−2 to 20)**	**Median: 8**	**Median: −2**
Proteinuria at ACEI cessation (mg/mmol creatinine)	Pt 1: 33, Pt 2: 30	Pt 1: 33	
	Pt 3: 21, Pt 4: 41	Pt 2: 30	
	Pt 5: 13	Pt 5: 13	Pt 3: 21
	Pt 6: 27, Pt 7: n.d.	Pt 6: 27, Pt 7: n.d.	Pt 4: 41
	**Median: 29 (13–41)**	**Median: 26**	**Median: 31**
Proteinuria post-ACEI cessation (mg/mmol creatinine)	Pt 1: 209	Pt 1: 209	
	Pt 2: 125	Pt 2: 125	
	Pt 3: 43, Pt 4: 175	Pt 5: n.d.	
	Pt 5: n.d.	Pt 6: 137	Pt 3: 43
	Pt 6: 137, Pt 7: 56	Pt 7: 56	Pt 4: 175
	**Median: 124 (43–209)**	**Median: 131**	**Median: 109**
Potassium at ACEI cessation (mmol/L)	Pt 1: 5.2	Pt 1: 5.2	
	Pt 2: 6.7	Pt 2: 6.7	
	Pt 3: 4.9	Pt 5: 5.4	
	Pt 4: 4.5, Pt 5: 5.4	Pt 6: 5.2	Pt 3: 4.9
	Pt 6: 5.2, Pt 7: 4.9	Pt 7: 4.9	Pt 4: 4.5
	**Median: 5.3 (4.5–6.7)**	**Median: 5.5**	**Median: 4.7**
Potassium post-ACEI cessation (mmol/L)	Pt 1: 4.4	Pt 1: 4.4	
	Pt 2: 5.0	Pt 2: 5.0	
	Pt 3: 5.2, Pt 4: 4.4	Pt 5: 4.3	
	Pt 5: 4.3	Pt 6: 4.9	Pt 3: 5.2
	Pt 6: 4.9, Pt 7: 3.5	Pt 7: 3.5	Pt 4: 4.4
	**Median: 4.5 (3.5–5.0)**	**Median: 4.4**	**Median: 4.8**
Blood pressure at ACEI cessation (mm Hg)	Pt 1: 136/68	Pt 1: 136/68	
	Pt 2: 107/66	Pt 2: 107/66	
	Pt 3: 104/68, Pt 4: 130/83	Pt 5: 102/60	
	Pt 5: 102/60	Pt 6: 106/72	Pt 3: 104/68
	Pt 6: 106/72, Pt 7: 96/64	Pt 7: 96/64	Pt 4: 130/83
	**Median: 106/68**	**Median: 106** **/** **66**	**Median: 117** **/** **76**
Blood pressure post-ACEI cessation (mm Hg)	Pt 1: 141/83	Pt 1: 141/83	
	Pt 2: 100/70	Pt 2: 100/70	
	Pt 3: 108/77, Pt 4: 134/103	Pt 5: 118/78	
	Pt 5: 118/78	Pt 6: 122/82	Pt 3: 108/77
	Pt 6: 122/82, Pt 7: 105/60	Pt 7: 105/60	Pt 4: 134/103
	**Median: 118/78**	**Median: 122** **/** **82**	**Median 121** **/** **90**

ACEIs, angiotensin-converting enzyme inhibitors; eGFR, estimated glomerular rate; PUV, posterior urethral valve; RN, reflux nephropathy bilateral; ARPKD, autosomal recessive polycystic kidney disease; NPHP, nephrophthisis.

The median values are indicated in bold.

## Discussion

In this small case series from our center, we compare the kidney function before and after withdrawal of ACEI in children with rapidly declining CKD stages 4–5. We could demonstrate that kidney function, similarly to the adults, improved also in children by approximately 70%.

There are at least three adult studies showing that the withdrawal of ACEI in CKD patients leads to improved GFR (2–4). Ahmed et al. demonstrated in an adult study that eGFR increased in 60% of patients with advanced CKD and undoubtedly delayed the onset of KRT in the majority of patients after stopping inhibitors of the RAAS (2). Hansen et al. observed increased GFR 1 month after withdrawal of long-term antihypertensive treatment, mainly ACEI, in 42 patients with diabetic nephropathy and CKD stages 1–2 (3). Onuigbo and Onuigbo demonstrated in their prospective study on 100 consecutive patients with advanced CKD stage 4 (mean GFR at stopping 22 ml/min) who presented with >25% increase in baseline that serum creatinine before enrollment (rapid progressors) that withdrawal of RAAS blockers lead to increase in GFR in 74% of patients (4). The median percentage of GFR improvement in the responders was 64%, again similar to the study done by Ahmed et al. However, Hsu et al. showed in their population-based observational study from Taiwan that the use of RAAS blockers in predialysis CKD stage 5 adult patients was associated with 6% lower risk for long-term dialysis (6).

On the contrary to the three adult studies that stopped ACEIs, the only pediatric study published recently by van den Belt et al. (1) showed in a retrospective design that discontinuation of RAASi in children with advanced CKD led to more rapid eGFR decline than before RAASi discontinuation. This finding is in sharp contrast to previous similar trials in adults.

The reasons for discrepant results in the study by van den Belt et al. (1) and others (2–4) and our case series could be several: eGFR at stopping of ACEIs, rate of the progression of CKD, reasons for stopping, BP, proteinuria, or type of study. In our patients and in the study by Ahmed et al., the eGFR at stopping ACEI was considerably lower than in the study by van den Belt et al. (12 and 16 vs. 27 ml/min/1.73 m^2^) (1, 2). Moreover, the eGFR slope before stopping ACEI was 4–7 times faster in our patients and the study by Ahmed et al. than in the study by van den Belt et al. (10 and 6 vs. 1.5 ml/min/1.73 m^2^/year) (1, 2). The children from the latter study would, therefore, progress to CKD stage 5 (GFR <15 ml/min/1.73 m^2^) very slowly and as late as in 8 years.

The eGFR slope after stopping ACEI in our study was +5 ml/min/1.73 m^2^/year (ranging −2 to 20, Table 1), which is in contrast to the results in the study by van Belt et al. where the eGFR slope increased from −1.5 to −3.9 ml/min/1.73 m^2^/year. The reasons for the different results in these two studies can be similar to that discussed earlier.

The reason for stopping ACEI was always the rapid decline of GFR toward CKD stage 5 in both studies with improvement of GFR after stopping. On the contrary, in the study by van den Belt et al., increase of serum creatinine was the reason for discontinuation in only 33% of patients; the remaining 67% had other reasons such as hyperkalemia (1). It would be interesting to know whether this subgroup of 33% children with increase of serum creatinine as the indication for discontinuation of RAASi did better than the whole group. Chan et al. hypothesized in their educational review on this topic that the study done by van den Belt et al. is limited by its observational nature and selection bias (7).

In another pediatric study, Abraham et al. (8) showed in children with CKD from the CKiD study that the use of RAASi was found to reduce the risk of KRT by 21% in comparison to nonusers. However, the median eGFR in discontinuers was much higher (26 ml/min/1.73 m^2^ with no patient <15 ml/min/1.73 m^2^) than in our case series (12 ml/min/1.73 m^2^), and the reasons for RAASi discontinuation were not specified. Furthermore the authors stated that “our results suggest that clinicians choose to maintain residual kidney function by discontinuing ACEi/ARBs.”

We speculate that the different results between our case series, adult studies, and those by van den Belt et al. and Abraham et al. are mainly due to different GFR at ACEI stopping (lower in the adult studies and our patients) and GFR slope before stopping (faster in the adult studies and our patients). We hypothesize, that highly selected patients with very low GFR at CKD stage 5 (10–15 ml/min/1.73 m^2^) being prepared for the start of KRT with a rapid declining GFR slope might potentially profit from stopping ACEI with subsequent improvement of GFR as it has been shown in adults and in our pediatric cases.

Our case series has strengths and limitations. The main strength is the fact that it is only the second pediatric study dealing with kidney effects of withdrawal of ACEIs in patients with advanced CKD. There are several limitations, especially very small number of cases, the retrospective design, and the lack of a control group of patients without ACEI withdrawal.

In a most recent article on the topic of stopping ACEIs in advanced CKD, Chan et al. recommended cessation of RAASi if patients develop rapidly declining kidney function progressing to CKD stage 5 (7). Nevertheless, only the results of the ongoing randomized controlled trial of RAAS blockers’ withdrawal in advanced renal disease (ongoing STOP-ACEi trial), will answer the question whether stopping ACEI in patients with advanced CKD is beneficial (9).

## Conclusion

Our case series suggests that in some children with advanced CKD stage 5 and rapidly deteriorating kidney function, stopping ACEIs may lead to an increase in eGFR in some patients.

## Data Availability

The original contributions presented in the study are included in the article, further inquiries can be directed to the corresponding author.
